# Potent Inhibition of miR-34b on Migration and Invasion in Metastatic Prostate Cancer Cells by Regulating the TGF-β Pathway

**DOI:** 10.3390/ijms18122762

**Published:** 2017-12-19

**Authors:** Li-li Fang, Bao-fei Sun, Li-rong Huang, Hai-bo Yuan, Shuo Zhang, Jing Chen, Zi-jiang Yu, Heng Luo

**Affiliations:** 1 State Key Laboratory of Functions and Applications of Medicinal Plants, Guizhou Medical University, Guiyang 550014, China; fanglili990538@163.com (L.-l.F.); sbf565912218@163.com (B.-f.S.); hlrong126@163.com (L.-r.H.); yhb717697@163.com (H.-b.Y.); shushuo@163.com (S.Z.); cj375307579@163.com (J.C.); 2 Key Laboratory of Chemistry for Natural Products of Guizhou Province and Chinese Academy of Sciences, Guiyang 550002, China; 3 Department of Anatomy, Guizhou Medical University, Guiyang 550000, China

**Keywords:** invasion, migration, miR-34b, prostate cancer, TGF-β signaling pathway

## Abstract

The importance of miRNAs in the progression of prostate cancer (PCa) has further been supported by the finding that miRNAs have been identified as potential oncogenes or tumor suppressors in PCa. Indeed, in eukaryotes, miRNAs have been found to regulate and control gene expression by degrading mRNA at the post-transcriptional level. In this study, we investigated the expression of miR-34 family members, miR-34b and miR-34c, in different PCa cell lines, and discussed the molecular mechanism of miR-34b in the invasion and migration of PCa cells in vitro. The difference analyses of the transcriptome between the DU145 and PC3 cell lines demonstrated that both miR-34b and -34c target critical pathways that are involved in metabolism, such as proliferation, and migration, and invasion. The molecular expression of miR-34b/c were lower in PC3 cells. Moreover, over-expression of miR-34b/c in PC3 cells caused profound phenotypic changes, including decreased cell proliferation, migration and invasion. Moreover, the players that regulate expression levels of transforming growth factor-β (TGF-β), TGF-β receptor 1 (TGF-βR1), and p53 or phosphorylation levels of mothers against decapentaplegic 3 (SMAD3) in the TGF-β/Smad3 signaling pathway have yet to be elucidated, and will provide novel tools for diagnosis and treatment of metastatic PCa.

## 1. Introduction

Prostate cancer (PCa) is the most common malignancy among men, and is one of the leading causes of cancer-related deaths in the United States [1]. In 2016, the incidence of PCa accounted for 21% of all cases in men, ranking first in the United States, and the mortality ranked second among all newly diagnosed cancer in men [1]. In the United States, over 180,890 new prostate cancer cases were diagnosed and approximately 26,120 died because of PCa in 2016 [1]. At present, early-stage PCa can be treated with prostatectomy and radiation therapy [2]. Androgen deprivation therapy is the standard treatment for advanced prostate cancer. However, almost all patients with lesions will gradually develop castration-resistant prostate cancer (CRPC), which is an incurable disease that develops after continued hormone therapy for one or two years. Therefore, currently no effective treatment options exist for metastatic or recurrent tumors [3]. Advanced PCa is often associated with metastatic characteristics, typically to the bones [4], and in 50–70% of castration-resistant prostate cancer patients bone metastases have been observed [5]. However, little is known about the molecular mechanisms of selective metastasis of PCa cells. In previous studies, it has been suggested that unique molecular signatures or biomarkers exist that predispose a PCa patient to metastasis. Activation of the rat sarcoma (Ras) signaling pathway is among the most commonly observed characteristics during metastasis in advanced PCa [6]. In a recent study, it was demonstated that bone metastasis and anti-apoptotic effects were found in Ras signaling-activated prostate cancer cells [7]. Upregulation of Ras-mediated signaling may be associated with various pathways, including the mitogen-activated protein kinase (MAPK) pathway [8], phosphatidylinositol 3-kinase (PI3K)/ protein kinase B (AKT) cascades [9], and WNT (named as a hybrid of Wingless and Int) signaling [10].

MicroRNAs (miRNAs) are a class of single-stranded non-coding small RNAs, approximately 22 nucleotides (nts) in length, that can bind target mRNAs via partial or complete complementary base pairing. MiRNAs regulate gene expression of oncogenes or tumor-suppressor genes at the post-transcriptional level to control a wide range of biological processes [11], such as cell cycle regulation, differentiation, metabolism, apoptosis, invasion, tumorigenesis, angiogenesis, and metastasis [12]. This suggests that miRNAs have become important mediators of cancer development and therapy. Previous studies have demonstrated that one single miRNA can regulate the expression of over two hundred target genes, and that the expression of certain target mRNAs can be regulated by several miRNAs [13]. Overall, more than one third of structural human genes resulted by regulation of miRNAs [14]. Considering their roles, it is not surprising that abnormal miRNA expression is related to several cancer pathologies, thereby making miRNAs potential clinical biomarkers of cancer patients. For example, miRNAs can be used as specific biomarkers for diagnosis, treatment, and prognosis of tumors [15]. Consequently, in many studies it has been demonstrated that miRNAs play an important role in the occurrence and development of cancer. Based on the above-mentioned new findings, the function and potent application of miRNAs have attracted increased interest in cancer biology.

Several miRNAs, such as miR-21, miR-34a, miR-203, miR-205 and miR-331, have been reported to affect cell proliferation, and metastasis in PCa. Up-regulation of miR-21 increased invasiveness of LNCaP PCa cells [16], however, several other miRNAs, such as miR-34a [16], miR-205 [17], and miR-16 [18], have been reported to inhibit the metastatic ability, and may act as targets of potential therapeutic agents for treating of metastatic PCa. The miRNA-34 family, a highly-conserved family among different species, is comprised of miR-34a, miR-34b and miR-34c. MiR-34a is located on chromosome 1p36, whereas miR-34b and miR-34c are clustered as homologous genes, and share a common primary transcript. In the human genome, miR-34b and miR-34c are located on chromosome 11q23 [19]. Loss of heterozygosity within the 11q23 region has been observed in several solid cancers, including breast cancer [20], lung cancer [21], and PCa [22]. Several studies have indicated that miR-34a and miR-34c, despite their seed regions being identical, exert different regulatory abilities due to their target genes [23,24]. Although the family has a homology of >80% and a share similar set of target genes, their different and important roles in carcinogenesis and the development of tumors need to be further investigated [25]. In a previous study, it was shown that miR-34a-c was a direct transcriptional target of tumor suppressor p53 [26]. In response to DNA damage, hypoxia, and oncogenic stress, p53 activates the miR-34 family of genes and induces cell cycle G1-arrest and/or apoptosis [27], inhibits cell proliferation, and epithelial to mesenchymal transition, migration, invasion, and metastasis of various cancer cell entities [28].

The Target Scan method showed that miR-34a, 34b, 34c and miR-449a can bind to mesenchymal-epithelial transition (MET) in PCa cells. Moreover, the migration of cancer cells was controlled by regulating miR-34c through the MET target in vitro and in vivo [29]. This will provide insight on predicting cancer cell migration using miRNAs as specific targets or biomarker in the diagnosis, treatment, and prognosis of advanced cancer. We observed a significant difference in miR-34b and miR-34c expression between the prostate cancer cell lines PC3 and DU145 (data are described in the “Results” section), however, PC3 and DU-145 are PCa cell lines with a moderate to high metastatic potential. This was based on their capacity to invade the extracellular matrix, an established tumor invasion assay, which indicated that expression of miR-34b and 34c correlated with the metastatic potential of PCa cells. An increasing number of studies have indicated that miR-34c may be involved in a novel molecular mechanism that uses miR-34c as a key tumor suppressor by targeting MET regulation in PCa. However, at present, the novel molecular mechanism of miR-34b has not yet been elucidated. In this study, we aimed to investigate the targets and preliminary molecular mechanism of action of miR-34b in migration and invasion of metastatic PCa cells. Using ectopic expression of miR-34b in vitro, followed by gene expression array, we found that the TGF-β signaling pathway was a target of miR-34b in PCa cells. Moreover, we demonstrated that members of TGF-β signal pathway, such as TGF-β, TGF-βR1 and p53 expression, and phosphorylation of mothers against decapentaplegic 3 (SMAD3) inversely correlated with miR-34b levels in PCa cells. Our working hypothesis was that miR-34b plays a role in the initiation, progression, metastases, and transition to castration resistance of PCa, and that some of effects may be mediated by targeting SMAD3 phosphorylation in the TGF-β signaling pathway.

## 2. Results

### 2.1. Analysis of the Metastatic Potential of Different Prostate Cancer Cells In Vitro

**Figure 1:**
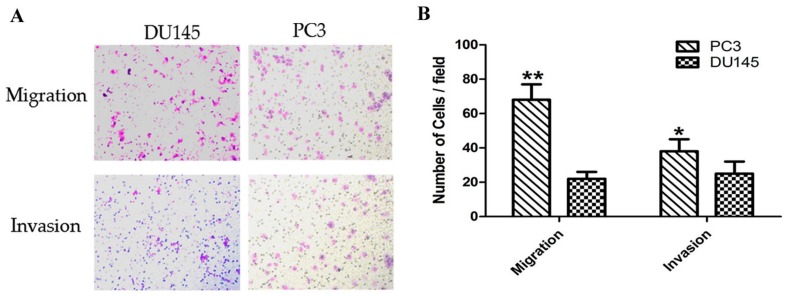
Comparison of migration and invasion capacity of prostate cancer cells. (**A**) Representative images of the invasion and migration of DU145 and PC3 cells taken by an inverted microscope (20× objective); (**B**) Quantitative analysis of cell migration (2 h) and invasion (4 h) in DU145 and PC3 cells. * *p* < 0.05 ** *p* < 0.01. Per condition, three independent experiments were performed.

Several studies have confirmed that DU145 and PC3 are PCa cell lines with moderate and high metastatic potential [30], respectively. In this study, we used the migration and invasion assay to demonstrate the metastatic capacity of the cells to invade the extracellular matrix. Thus, we established a tumor invasion assay to verify the metastatic capacity of androgen-independent PCa cells. The data showed that in PC3 cells, the number of migrating and invading cells increased by 3-fold (*p* < 0.01) and 1.5-fold (*p* < 0.05) when compared to that of DU145 cells (Figure 1). Moreover, the results indicated that DU145 and PC3 are PCa cell lines with a moderate and high metastatic potential, respectively. This suggested that the cultured cells were suitable for use in other experiments.

### 2.2. Expression of miR-34 Family Members in Different Metastatic Potential Prostate Cancer Cells

**Figure 2:**
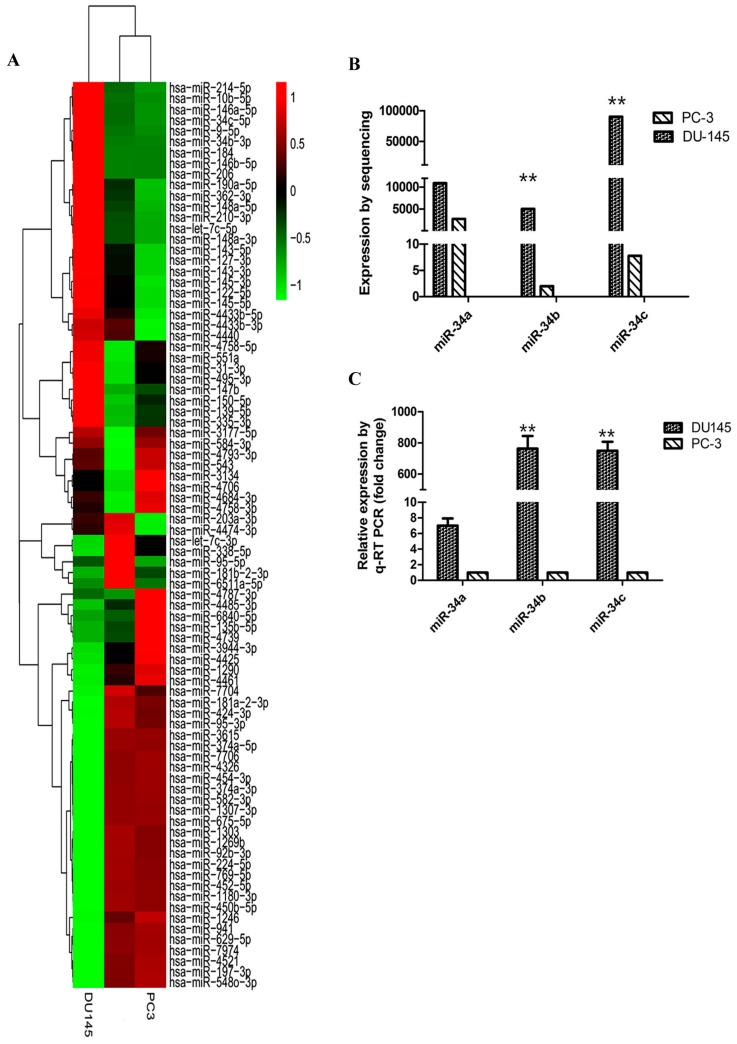
Expression levels of miR-34a, miR-34b, and miR-34c in prostate cancer cell lines DU145 and PC3. (**A**) Heat-map of miRNAs with differential expression comparing DU145 and PC3 cells. Up-regulated miRNAs are in red, whereas down-regulated genes are shown in green. Expression of miR-34b and miR-34c is up-regulated in DU145 cells and downregulated in PC3 cells; (**B**) Expression of miR-34a, miR-34b and miR-34c in DU145 and PC3 cells by miRNA sequencing analysis; (**C**) The relative expression of miR-34a, miR-34b, and miR-34c in DU145 and PC3 cells by qRT-PCR analysis. ** *p* < 0.01. Per condition, three independent experiments were performed.

To identify the differential expression of miRNAs in PCa cells, miRNA sequencing analysis was performed in DU145 and PC3 cells. The miRNAs expression profile in DU145 and PC3 cells by heat map analysis indicated that expression of miR-34b and miR-34c in DU145 cells were significantly higher compared to that in PC3 cells (Figure 2A). The expression level of three members of the miR-34 family was investigated in DU145 and PC3 cells using miRNA sequencing (Figure 2B) and by qRT-PCR (Figure 2C). MiRNA sequencing analysis demonstrated that miR-34b was 5000-fold higher expressed in DU145 cells (*p* < 0.01) compared to that in PC3 cells, whereas for miR-34a, the expression was only 4-fold higher. Therefore, our data showed that there was no significant difference in the expression level of miR-34a between PC3 and DU145 cells. We also determined that miR-34c had a higher level of expression in DU145 cells and was about 10,000-fold higher expressed compared to that in PC3 cells. These findings were consisted with the results of a previous report [31,32]. To confirm these findings, we evaluated the expression of miR-34 family members in two cancer cell lines using qRT-PCR analysis. We found that the expression level of three members of the miR-34 family showed the same trend that was found by miRNA sequencing, suggesting that miR-34b and miR-34c may be involved in prostate cancer metastasis. Our data demonstrated that the expression level of miR-34b and miR-34c negatively correlated with the metastatic potential in PCa. Moreover, our findings were in line with the wound healing assay results of a previous study [32].

### 2.3. Effects of miR-34b and miR-34c on Cell Viability and Proliferation of Prostate Cancer Cells

**Figure 3:**
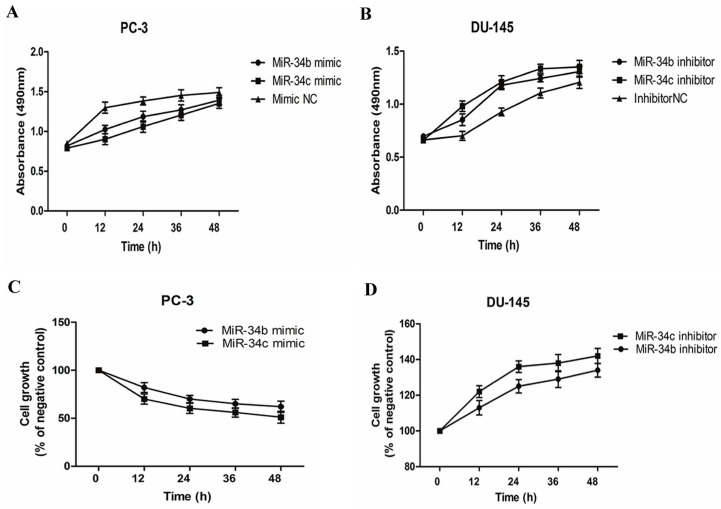
Both miR-34b and miR-34c suppresses prostate cancer cell proliferation. (**A**,**B**) 3-(4,5-dimethyl-2-thiazolyl)-2,5-diphenyl-2-H-tetrazolium bromide (MTT) assay of PC3 cells transfected with miR-34b/c mimic (50 nM) or mimic negative control (NC) (50 nM) and DU145 cells transfected with miR-34b/c inhibitor (100 nM) or inhibitor NC (100 nM); (**C**,**D**) Effects on cell growth with ectopic expression of miR-34b/c in DU145 and PC3 by live cells counting compared to the negative control. Per condition, three independent experiments were performed.

**Figure 4:**
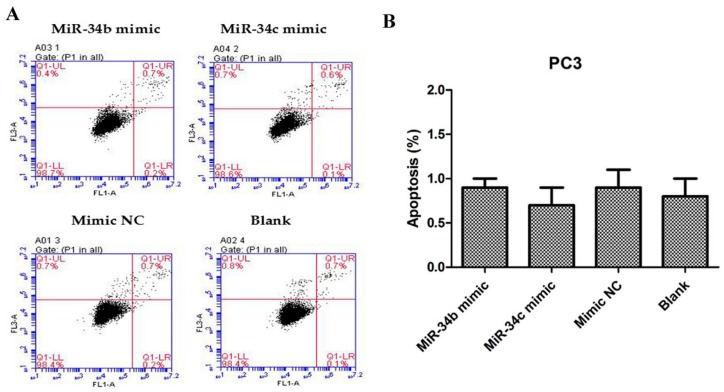
Flow cytometric data of apoptotic PC3 cells, 48 h after transfection with miR-34b/c mimic, mimic NC or without transfection (Blank). (**A**) Cells were harvested, stained with annexin-V-FITC and PI, and analyzed by flow cytometry. (**B**) Data showing the percentages of early and late apoptotic cells (right quadrants).

To investigate whether ectopic expression of miR-34b and miR-34c affects cell proliferation and growth in different PCa lines, 3-(4,5-dimethyl-2-thiazolyl)-2,5-diphenyl-2-H-tetrazolium bromide (MTT) assays were performed and live cells were counted after trypan blue staining. PC3 cells transfected with miR-34b/c mimic revealed pronounced growth inhibition (*p* < 0.05) compared with cells that were transfected with a mimic negative control (NC) (Figure 3A,C). DU145 cells transfected with miR-34b/c inhibitor showed significant growth promotion (*p* < 0.05) when compared with cells transfected with inhibitor negative control (NC) (Figure 3B,D). However, miR-34b and -34c did not induce apoptosis (Figure 4). Thus, these results demonstrated that miR-34b and miR-34c suppressed PCa cell proliferation.

### 2.4. Effects of Over-Expression of miR-34b and miR-34c on Migration and Invasion of PC3 Cells

**Figure 5:**
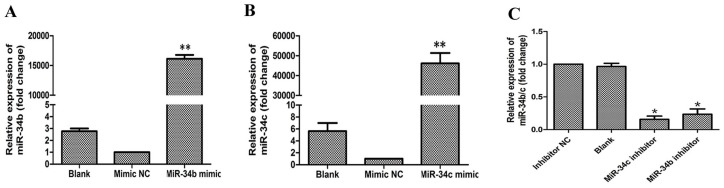
(**A**,**B**) Relative expression of miR-34b and miR-34c in PC3 cells transfected with miR-34b/c mimic or mimic negative control (NC) or without transfection (Blank) by qRT-PCR analysis. ** *p* < 0.01 vs. mimic NC or blank group; (**C**) qRT-PCR analysis showing the relative expression of miR-34b and miR-34c in DU-145 cells transfected with miR-34b/c inhibitor or inhibitor NC or without transfection (Blank). * *p* < 0.05 vs. inhibitor NC or blank group.

**Figure 6:**
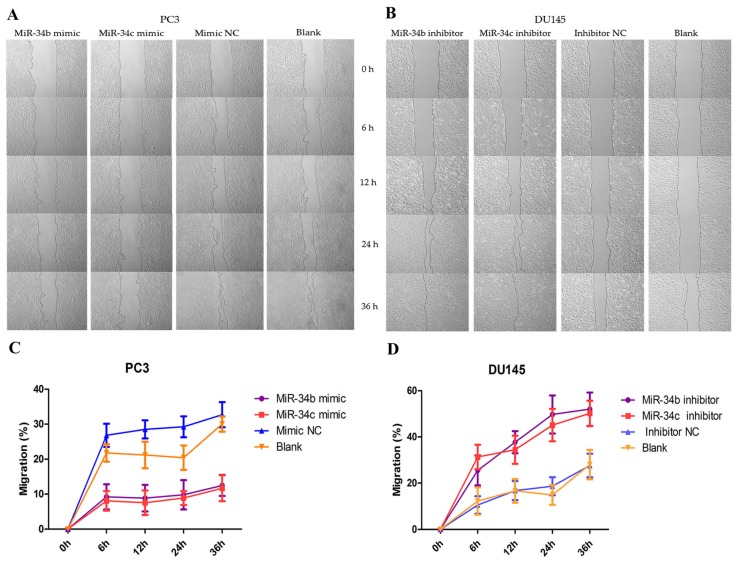
Random migration by a wound healing/scratch assay. (**A**,**B**) Shown are representative images of the scratch assay in DU145 and PC3 cells at 0, 6, 12, 24 and 36 h after the scratch was created; (**C**,**D**) The rate of migration calculated by the width of the wound in DU145 and PC3 cells after ectopic expression of miR-34b/c by Image-Pro Plus software (Media Cybernetics, MD, USA).

**Figure 7:**
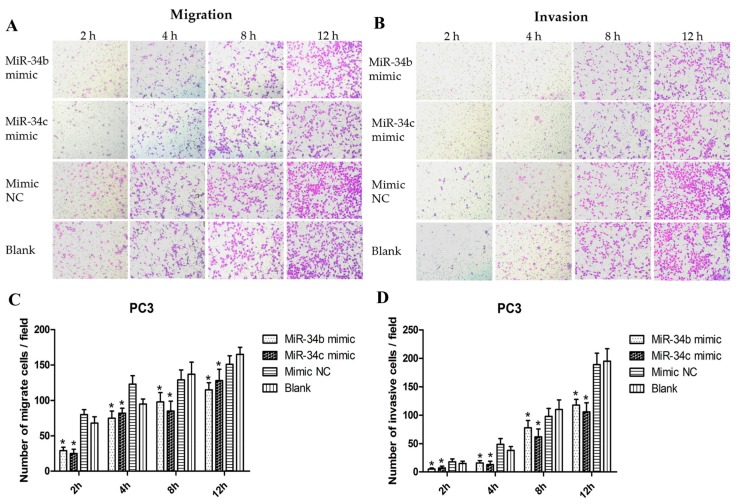
The capacity of migration and invasion of PC3 cells after transfection with miR-34b/c at 2, 4, 8, 12 h respectively. (**A**,**B**) Representative images of the invasion and migration of PC3 cells taken by an inverted microscope (20× objective); (**C**,**D**) Quantitative analysis of cell migration and invasion. * *p* < 0.05 vs. mimic NC or blank group (basal migration without transfection). Per condition, three independent experiments were performed.

To investigate the role of miR-34b and miR-34c in ability of PCa metastasis in vitro, wound healing/scratch assays and transwell migration and invasion assays were performed. PC-3 cells were transfected with miR-34b and miR-34c, after which the relative expression of miR-34b and miR-34c as well as of miR-34b/c mimic or mimic NC were detected by qRT-PCR (Figure 5A,B). PC3 cells that were transfected with miR-34b/c mimic were significantly reduced in the wound healing and transwell assays compared with that of the control (Figure 6 and Figure 7). These results demonstrated that over-expression of miR-34b and miR-34c reduced the migration and invasion ability in higher metastatic PC3 cells.

### 2.5. Effects of Inhibition of miR-34b and miR-34c on Migration and Invasion of DU145 Cells

**Figure 8:**
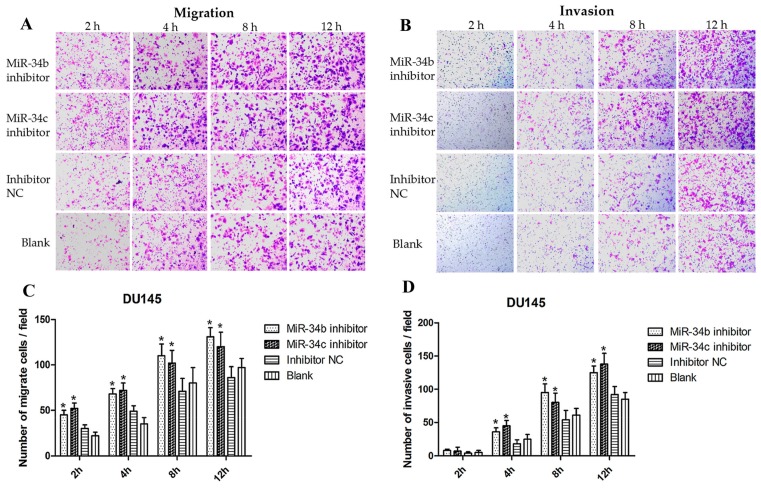
The migration and invasion capacity after transfection with miR-34b/c in DU-145 cells at 2, 4, 8, 12 h respectively. (**A**,**B**) Representative images showing the invasion and migration of DU-145 cells transfected with miR-34b/c inhibitor or inhibitor NC or without transfection (20× objective); (**C**,**D**) Quantitative analysis of cell migration and invasion. * *p* < 0.05 vs. inhibitor NC or blank group. Per condition, three independent experiments were performed.

To confirm the effects of miR-34b and miR-34c on the migration and invasion ability of PCa cells, DU145 cells were transfected with an miR-34b/c or inhibitor NC and analyzed by qRT-PCR (Figure 5C). The results indicated that the relative expression of miR-34b and miR-34c after transfection with the miR-34b/c inhibitor was decreased 5-fold compared with the inhibitor NC. However, the concentration of the miR-34b/c inhibitor used in our study, influenced to a small extent the inhibition of miR-34b and miR-34c expression. Thus, the wound healing and transwell assay indicated that the number of migrating and invading cells that were transfected with the miR-34b/c inhibitor were slightly increased (*p* < 0.05) (Figure 8), suggesting that inhibition of miR-34b and miR-34c expression slightly promoted the migration and invasion ability in low metastatic DU145 cells.

### 2.6. Analysis of Signaling Pathways Involved in Target Genes of miR-34b in Prostate Cancer Cells

**Figure 9:**
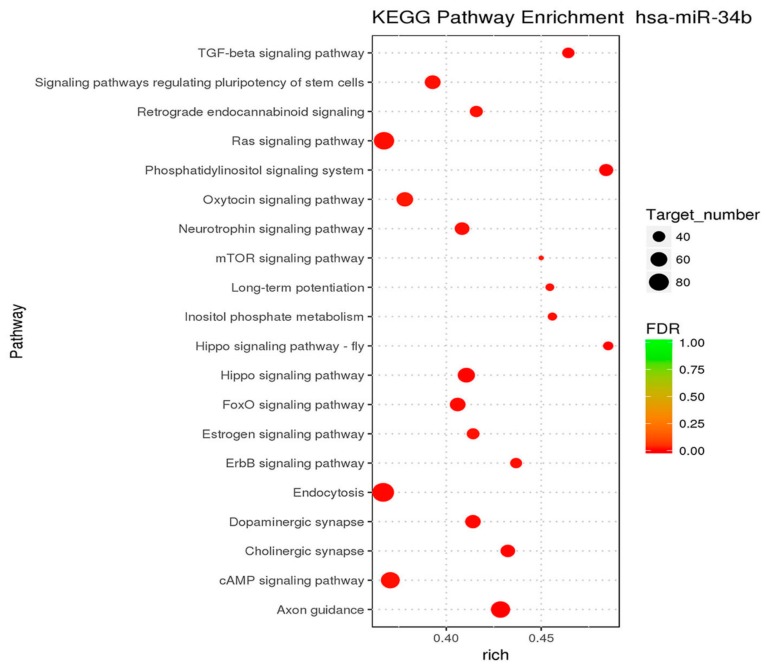
Kyoto Encyclopedia of Genes and Genomes (KEGG) enrichment analysis indicating the pathways affected by miR-34b in prostate cancer cells. The size of the dots indicates the number of differential genes in the pathway, whereas colors represent the significant *p*-value of the pathway. The 20 most significantly different pathways are shown.

To analyze the signal pathways that are involved in miR-34 family members, mRNA sequencing was performed using PC3 and DU145 cells. The target genes of miR-34b in different pathways were analyzed using Kyoto Encyclopedia of Genes and Genomes (KEGG) enrichment (Table 1). Hypergeometric distribution was used for calculating the target gene enrichment pathways (Figure 9), which showed that the main signaling pathways involved in the target genes of miR-34b included: TGF-beta signaling pathway, axon guidance pathway, regulation of actin cytoskeleton pathway, MAPK signaling pathway, vascular smooth muscle contraction, tight junction pathway, oxytocin signaling pathway, osteoclast differentiation, hippo signaling pathway, adherens junction pathway, apoptosis pathway, and ErbB signaling pathway. In this study, we focused on the TGF-β signaling pathway to gain better insight and understanding the function of miR-34b.

### 2.7. Regulations of miR-34b on TGF-β Signaling in PC3 Cells

**Figure 10:**
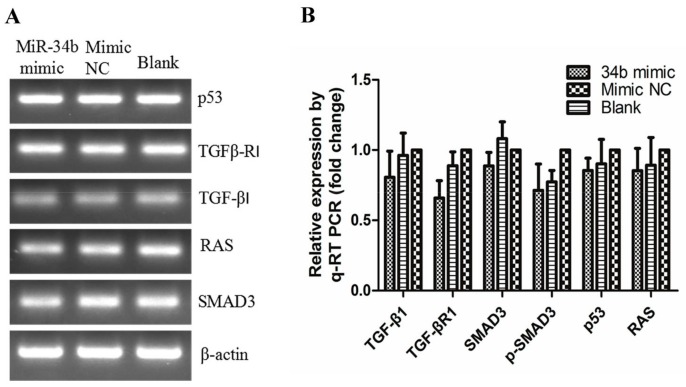
Transcript levels of TGF-β1, TGFβ-R1, SMAD3, p53 and RAS after treatment with 50 nM miR-34b mimic, NC mimic in PC3 cells by PCR (**A**) and qRT-PCR (**B**) β-actin was used as an internal control. The mRNA expression of genes was not significantly different in PC3 cells treated with or without miR-34b mimics. Per condition, three independent experiments were performed. TGF: transforming growth factor; SMAD: mothers against decapentaplegic; p53: a phosphorylated protein with a molecular weight of 53,000; RAS: rat sarcoma.

**Figure 11:**
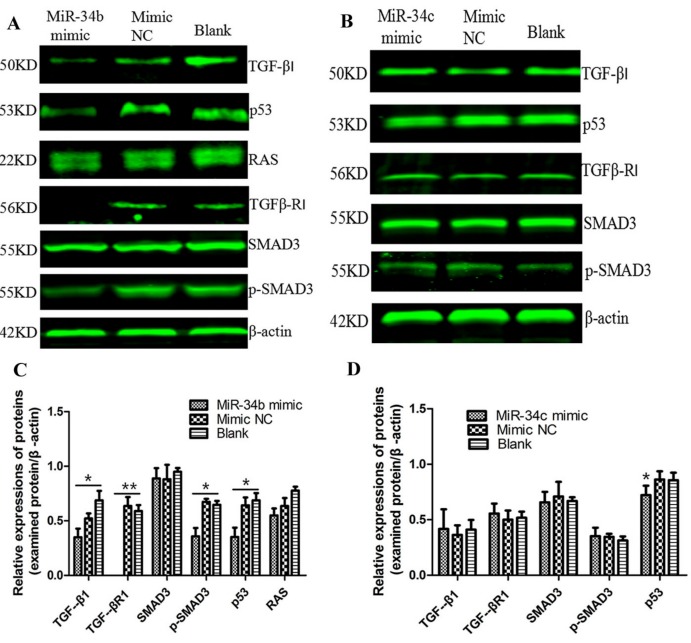
Western blot analysis of TGF-β1, TGFβ-R1, SMAD3, p-SMAD3, p53, and RAS after ectopic expression of miR-34b/c in PC3 cells. (**A**) Protein levels of TGF-β1, TGFβ-R1, and p53 and phosphorylation of SMAD3 in miR-34b mimic-treated PC3 cells were lower compared to that in mimic NC or blank control groups. Protein levels of RAS were not significantly different between groups; (**B**) In miR-34c mimic-treated PC3 cells, protein levels of TGF-β, TGFβ-R, p53 and phosphorylation of SMAD3 were not significantly different when compared to control groups; (**C**,**D**) The relative protein levels were calculated by Image J software (Rawak Software, Inc., Dresden, Germany), and β-actin was used as a loading control. ** *p* < 0.01 * *p* < 0.05 vs. mimic NC or blank group. Per condition, three independent experiments were performed.

PC3 cells were transfected with miR-34b/c mimic and mimic NC, and cultured for 48 h. Next, we used PCR analysis to evaluate at the transcript level the expression of members of the TGF-β signaling pathway, including TGF-β, TGF-βR1, SMAD3, p53 and RAS (Figure 10). Our data indicated that the expression of all key factors in the TGF-β signaling pathway did not show an obvious difference at the mRNA level, indicating that miR-34b did not regulate the TGF-β signaling pathway at the transcript level. Therefore, we further investigated the expression and phosphorylation level of key players in the TGF-β signaling pathway at the protein level by Western blot analysis (Figure 11). The results showed that miR-34b mimics significantly inhibited expression of TGF-β (*p* < 0.05), TGF-βR1 (*p* < 0.01), and p-SMAD3 (*p* < 0.05) at the protein level, however they had no effect on miR-34c-treated PC3 cells. The protein level of p53 was lower in both miR-34b and miR-34c-treated PC3 cells compared to the control group (*p* < 0.05). There was a limited effect on RAS, indicating that miR-34b may have suppressed the expression of TGF-β, TGF-βR1 in PCa at the post-transcriptional level.

## 3. Discussion

In this study, the mechanisms and implications of miR-34b in metastasis of PCa were investigated using a series of molecular and biochemical approaches. The difference in analyses of the transcriptome between DU145 and PC3 cells showed that miR-34b and miR-34c targeted key metabolic pathways, such as proliferation, migration, and invasion, but not apoptosis. These results were consisted with the effects of miR-34c on proliferation, apoptosis, migration, and invasion that were presented in previous studies, and correlated with clinical parameters, such as aggressiveness, occurrence of metastases, and overall survival in PCa patients [32]. The molecular expression was evaluated in PC3 cells, because of their low levels of miR-34b and because over-expression of miR-34b in PC3 cells gave profound phenotypic changes, including decreased cell growth, migration and invasion. Thus, the targets regulating the expression of TGF-β, TGF-βR1 and p53 or the phosphorylation level of SMAD3 in the TGF-β signaling pathway have yet to be explained, and will increase our understanding and provide novel tools for diagnosis and treatment of metastatic PCa.

Metastases are the main cause of cancer-related deaths, especially in PCa [33]. Accordingly, identifying the underlying molecular mechanism and discovering novel methods for preventing the metastasis of cancer cells are of critical importance for clinical diagnosis and treatment of various cancer types. The communication between tumor and stromal cells is mediated by secreted cytokines, which regulates every cell within the tumor microenvironment [34]. Moreover, a variety of growth promoting factors such as vascular endothelial growth factor (VEGF), TGF-β, and interleukin-8 (IL-8) actively participated in the induction of angiogenesis [35]. TGF-β is a multi-functional secreted cytokine that plays a variety of biological effects in physiological and pathological conditions [36]. Activated TGF-β binds to its receptor and exerts biological effects through SMAD or non-SMAD signaling pathways [37]. Recent studies have revealed that TGF-β promoted cancer metastatic dissemination by destruction of the extracellular matrix and angiogenesis, as well as by promoting immune evasion [34]. The current study aimed to elucidate whether miR-34b affected cell growth, migration, and invasion in higher metastatic PCa, and to investigate the possible underlying molecular mechanisms of action.

**Figure 12:**
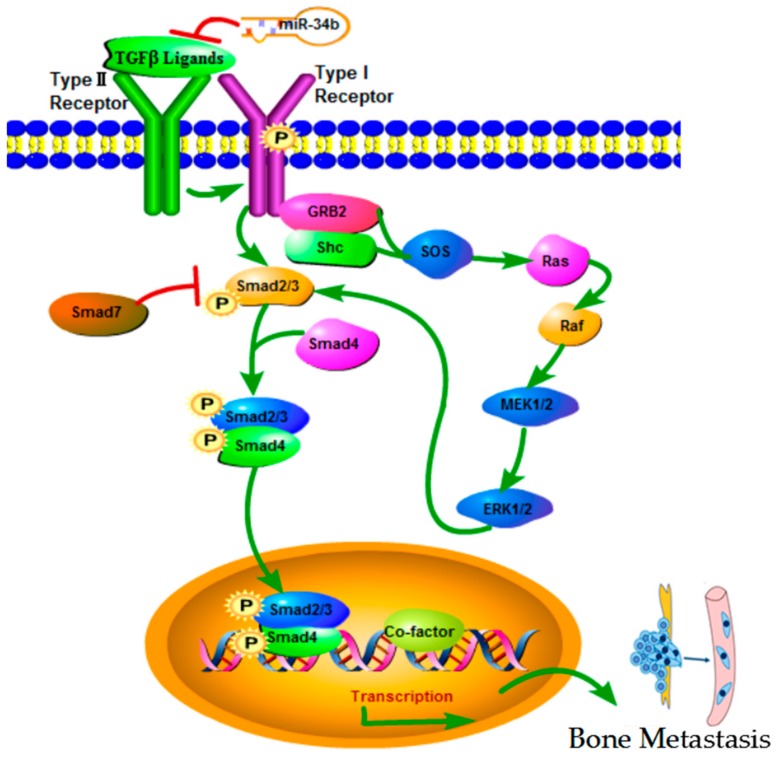
TGF-β signaling pathway. TGF-β superfamily of cytokines bind to type I receptors and type II receptors at the cell surface, thereby forming a tetrameric complex. Binding of TGF-β1 to its receptor II activated the TGF-β receptor type I kinase, resulting in phosphorylation of the downstream signal transducer Smad2/3(R-SMADs). Phosphorylated R-SMADs forms a complex with a common mediator, Smad4 (Co-SMAD). This complex is translocated into the nucleus, where it binds DNA and regulates transcription of many genes by interacting with transcriptional cofactors. SMAD7 represses signaling by other SMADs to down-regulate the system. Other signaling pathways, including the MAPK-ERK cascade are activated by TGF-β signaling, and modulate SMAD activation.

There were different mechanisms have discussed the role of miR-34 family members in regulating the tumorigenesis and development, including modulation of cell cycle transitions, EMT, metastasis, or cancer stemness [38]. For example, the report shown that loss of miR-34b is associated with progression of prostate cancer, and can accurately discriminate between benign hyperplasia and PIN lesions or infiltrating prostatic adenocarcinoma in humans, and identified the deregulation of MiR-34b/SRY-related high-mobility group box 2 (Sox2) may be as the biomarker for predicting prostate cancer progression [39]. However, TGF-β signaling pathway involved the regulation of miR-34 family members have no obvious conclusion in the present studies. TGF-β signaling pathway has been shown to be a key carcinogenic factor, and involves two receptors (I and II) and the SMAD family in cell signal transduction. TGF-βR1 can directly activate and phosphorylate SMAD2 and SMAD3 (R-SMAD), which bind to a SMAD4 (Co-SMAD) mediator to translocate into the nucleus and form complexes that regulate transcription [40] (Figure 12). TGF-βR1 was reported as a direct target for miR-140-5p, which inhibited TGF-β and MAPK/extracellular signal-regulated kinase (MAPK/ERK) signaling to suppresses tumor growth and metastasis in hepatocellular carcinoma [41]. Wu et al. [42] demonstrated that in colon cancer, miR-193b affected cell growth through the TGF-β and SMAD3 signaling pathways. However, no reports are available that focus on whether miR-34b suppresses metastases through the TGF-β/SMAD3 pathway. Our findings showed that over-expression of miR-34b downregulated the expression of TGF-βR1 and the phosphorylation of SMAD3 at the protein level, without affecting the mRNA levels. These findings were in agreement with previous reports that showed that miRNA inhibited target gene expression post-transcriptionally [19], thereby indicating that miR-34b suppressed the migration and invasion of PCa via TGF-βR1.

In summary, our study was the first time to provide evidence that the TGF-β signaling pathway may be a key target of miR-34b to regulate cell migration and invasion of PCa. In our study, we found that phosphorylation of SMAD is observed during metastasis of advanced PCa. In addition, activation of Ras signaling is widely observed during metastasis of advanced PCa. Therefore, the TGF-β/SMAD3 pathway is a key regulatory approach of miR-34b in regulating migration and invasion of higher metastatic PCa. Our results will give insight information on investigating the role of miR-34b through regulating the TGF-β signal pathway in PCa cells for treating metastatic PCa.

## 4. Materials and Methods

### 4.1. Prostate Cancer Cell Lines

The human prostate cancer cell lines PC3 and DU145 were a gift from the Sunnybrook Research Center (Toronto, ON, Canada). Cells were cultured in Dulbecco’s Modified Eagle’s Medium (DMEM) (Hyclone, South Logan, UT, USA), supplemented with 10% fetal bovine serum (FBS) and 1% penicillin and streptomycin (Sijiqing, Hangzhou, China) at 37 °C and 5% CO_2_.

### 4.2. RNA Extraction and Reverse Transcription

For determination of miRNA and mRNA levels, total RNA was isolated from PCa cells using TRIzol^®^ reagent (Invitrogen, Carlsbad, CA, USA). A total of 5 × 10^6^ cells were lysed in TRIzol reagent and centrifuged for 15 min at 12,000× *g* and 4 °C. Then, chloroform was added, and the mixture was allowed to separate into a clear upper aqueous layer, containing RNA. The RNA was precipitated with isopropanol, washed with 75% ethanol, and diluted in RNase-free water. All steps were performed on ice. The A260/A280 ratio and the RNA concentration were measured using a NanoDrop 2000 apparatus (Thermo Scientific, Waltham, MA, USA), and the RNA quality was determined by agarose gel electrophoresis. Total RNA (4500 ng) was then reverse-transcribed into cDNA using a HiFiScript cDNA Synthesis Kit (CWBIO, Beijing, China) with miRNA-specific RT primers (Ribobio, Guangzhou, China) for miR-34a, miR-34b and miR-34c according to the manufacturer’s guidelines. Reverse transcription without RT primers was used for detection of the gene expression of TGF-β1, TGFβ-R1, SMAD3, p53 and RAS.

### 4.3. MicroRNA Sequencing and Analysis

Sequencing of microRNA was performed to determine the microRNA profile of PC3 and DU145 cells. In brief, small RNA libraries were constructed using the NEBNext Multiplex Small RNA Library Prep Set for Illumina according to the manufacturer’s guidelines. The library was sequenced on a Hiseq platform (Illumina) by Shanghai Personal Biotechnology Co., Ltd. (Shanghai, China). DESeq (version 1.18.0, Anders S and Huber W, 2010, Heidelberg, Germany) was used to analyze differential expression of miRNAs, and differential mature miRNA was screened based on multiple differences (|fold change| > 2), and significant difference in expression (*p*-value < 0.05). Pathways affected by miR-34 in PCa cells underwent knockout (KO) enrichment analysis. By using the genome as the background, pathways of the target were significantly enriched and were calculated by hypergeometric distribution.

### 4.4. Real-Time Quantitative RT-PCR (qRT-PCR)

Real-Time Quantitative RT-PCR (qRT-PCR) amplification was performed using stem-loop primers for miR-34 designed by Ribobio (Guangzhou, China), primers for TGF-β1, TGFβ-R1, SMAD3, p53, and RAS (Sangon Biotech, Shanghai, China), and UItraSYBR Green qPCR Mixture (with ROX) reagents (CWBIO, Beijing, China) in a StepOnePlus™ Real-Time PCR System (Thermo Fisher Scientific, Waltham, MA, USA) using the following protocol: 10 min at 95 °C for the initial denaturation, followed by 95 °C for 15 s for 40 cycles, 60 °C for 1 min at the cycling stage, and 95 °C for 15 s, 60 °C for 1 min, 95 °C for 15 s at the melt curve stage. β-actin served as an endogenous control, and the 2^−ΔΔ*C*_T_^ method was used to calculate relative expression levels. Primer sequences are shown in Table 2.

### 4.5. Cellular Transfection

Cells were seeded at a density of 6 × 10^5^ cells in 60-mm dishes, and grown to ~80% confluency for transfection. PC-3 and DU-145 cells were transfected with miR-34b/c mimic (50 nM; Ribobio, Guangzhou, China) or miR-34b/c inhibitor (100 nM; Ribobio, Guangzhou, China) using Lipofectamine^®^ 3000 reagent (Invitrogen, Carlsbad, CA, USA). MicroRNA mimic or inhibitor in 250 μL of Opti-MEM^®^ (Gibco, Grand Island, NY, USA) medium was added to 10 μL of Lipofectamine in 250 μL of Opti-MEM^®^ medium and incubated for 20 min at room temperature. Cells were maintained in 3 mL of Opti-MEM^®^ medium containing the complexes for 12 h at 37 °C, then, medium was removed and replaced with serum-supplemented medium and cultured for another 36 h. After transfection for 48 h, cells were harvested and the transfection efficiency was determined by qRT-PCR. In this study, mimic or inhibitor-negative control (NC, Ribobio, China) was used as a control for non-sequence-specific effects in miRNA experiments.

### 4.6. Cell Proliferation Assay

Cells (0.5 × 10^4^) transfected with miRNA for 48 h were seeded into 96-well plates and incubated at 37 °C for 12 h, 24 h, 36 h and 48 h, respectively. Then, 10 µL of MTT solution (5 mg/mL) was added to each well and incubated for 4 h at 37 °C. After centrifugation at 2500 rpm/min for 15 min, medium was removed and 150 µL of DMSO was added to each well. The plated was incubated for 15 min at room temperature and the absorbance was measured at 490 nm using a Synergy2 microplate assay reader (BioTek Instruments, Winooski, VT, USA).

### 4.7. Cell Apoptosis Assay

Apoptosis was evaluated microscopically and by a flow cytometry-based assay using the annexin V-fluorescein isothiocyanate (FITC) and propidium iodide (PI) staining kit (BD Pharmingen, San Diego, CA, USA). Apoptotic cells were indicated as annexin-V-positive. Cells were trypsinized, washed twice with PBS and centrifuged at 1000 rpm for 5 min at room temperature. Then, cells were resuspended in 1× binding buffer, 5 μL of PI and FITC were added per 1 × 10^5^ cells, cells were vortexed, and incubated for 15 min at room temperature in dark. Subsequently, cells were analyzed by flow cytometry (Becton Dickinson, Franklin Lakes, NJ, USA).

### 4.8. Wound Healing Assay

To assess cell motility, an in vitro wound healing/scratch assay was performed. PC3 and DU145 cells were transfected with miR-34b/c mimic, a negative control or an inhibitor, respectively, and allowed to grow to confluence. Then, a scratch was made in the monolayer with a sterile micropipette tip. The cell layer was washed twice with culture media and incubated for 6, 12, 24 and 36 h with Opti-MEM^®^ medium (Gibco, Grand Island, NY, USA). The size of the scratch, from 3 independent experiments, was measured at 5 random sites in images taken using a Camedia C-5050 camera (Olympus Tokyo, Japan) and the data were analyzed by Image-Pro Plus software (Media Cybernetics, Rockville, MD, USA).

### 4.9. Migration and Invasion Assays

The migration and invasion capacity of the cells was assessed by transwell migration and invasion assays using transwell chambers (8.0 μm pore size, Corning, Corning, NY, USA) coated without or with Matrigel (BD Biosciences, Franklin Lakes, NJ, USA) diluted to 3 mg/mL using ice-cold Opti-MEM^®^ medium. The chambers were placed in 300 μL diluted Matrigel solution and incubated for 30 min at 37 °C for the “gelling” process to complete. Then, 200 μL (5 × 10^4^ cells) of PC-3 and DU-145 cells transfected with miR-34b mimics, inhibitor, or negative control for 48 h were resuspended in Opti-MEM^®^ medium and plated into the upper chamber of the transwell without or with Matrigel. For cell migration analysis, DMEM supplemented with 10% FBS was added into the lower chamber of the transwell, however, for invasion assays, DMEM was supplemented with 20% FBS. After 2, 4, 8, 12 h of incubation at 37 °C and 5% CO_2_, invasive and migrated cells that transferred through the membrane to the lower surface were fixed in 75% ethylalcohol, stained with 0.1% crystal violet, and counted in five visual fields of three independent experiments using an inverted microscope (Nikon, Tokyo, Japan) and a 20× objective after.

### 4.10. Western Blot Analysis

Transfected cells were lysed in RIPA buffer containing 1% protease inhibitor AEBSF (LanMu, Shanghai, China) for 30 min, centrifuged at 12,000 rpm for 15 min at 4 °C, and the protein concentrations were determined using the Pierce Bicinchoninic acid (BCA) Protein Assay Kit (Beyotime, Shanghai, China). Total proteins were separated by SDS-PAGE (the concentration was based on the molecular weight of the antibody), and transferred to polyvinylidene fluoride (PVDF) membranes (0.2 μm, Merck KGaA, Darmstadt, Germany). Membranes were blocked in the buffer containing 5% non-fat milk in Tris-buffered saline, containing 0.1% Tween-20 (TBST) for 1h and incubated overnight with the following primary antibodies: rabbit monoclonal anti-TGFβRI (ab135814) (1:100), anti-SMAD3 (ab40854), anti-p-SMAD3 (phospho S423 + S425) (ab52903), anti-RAS (ab52939), anti-p53 (ab75754), and anti-TGF-βI (ab179695) (1:1000) at 4 °C. Membranes were washed with TBST, and incubated with goat anti-rabbit IgG H&L (ab97047) secondary antibodies (1:30,000). A rabbit polyclonal to β-actin antibody (ab119716) was used as a loading control. The straps were detected by an Odyssey Infrared Imaging System and relative protein levels of the genes involved were measured by the gray-scale value of straps using Image J software.

### 4.11. Statistical Analysis

Data obtained from the qRT-PCR on miR-34 expression and the ability of migration and invasion in PC-3 and DU145 cells, were analyzed using a Student’s *t*-test from at least 3 independent experiments. For the other data, One Way ANOVA was used and *p* < 0.05 was considered statistically significant. * *p* < 0.05, ** *p* < 0.01.

## Figures and Tables

**Table 1:** Signaling pathways involved in miR-34b function.

Pathway	miR-34b Target up Number ^1^	DEG Number ^2^	Total Number ^3^	*p*-Value
TGF-beta signaling pathway	12	12	66	0.00123255
Axon guidance	29	29	165	0.000272805
Regulation of actin cytoskeleton	28	28	192	0.006013617
MAPK signaling pathway	31	31	238	0.01931642
Vascular smooth muscle contraction	18	18	117	0.01461593
Tight junction	19	19	130	0.020507
Oxytocin signaling pathway	22	22	146	0.009535646
Osteoclast differentiation	21	21	122	0.002336558
Hippo signaling pathway	21	21	133	0.006598796
Adherens junction	15	15	70	0.001029512
Apoptosis	19	19	129	0.01903818
ErbB signaling pathway	14	14	83	0.01381979

^1^ The number of genes upregulated by miR-34b; ^2^ number of differentially expressed genes; ^3^ number of genes annotated by genomes enriched in the pathways (*p* < 0.05). DEG: differentially expressed genes; TGF: transforming growth factor; MAPK: mitogen-activated protein kinase; ErbB: Erythroblastic Leukemia Viral Oncogene Homolog.

**Table 2:** Primer sequences of the genes used in qRT-PCR.

Gene	Primer	Primer Sequence (5′-3′)
TGFBR1	Forward	ACGGCGTTACAGTGTTTCTG
	Reverse	GCACATACAAACGGCCTATCTC
KRAS	Forward	ACAGAGAGTGGAGGATGCTTT
	Reverse	TTTCACACAGCCAGGAGTCTT
SMAD3	Forward	TGGACGCAGGTTCTCCAAAC
	Reverse	CCGGCTCGCAGTAGGTAAC
TGFB1	Forward	GGCCAGATCCTGTCCAAGC
	Reverse	GTGGGTTTCCACCATTAGCAC
β-actin	Forward	GCCAACACAGTGCTGTCT
	Reverse	AGGAGCAATGATCTTGATCTT

## Abbreviations

TGF-β	Transforming growth factor-β
TGF-βR	TGF-β receptor
SMAD3	Mothers against decapentaplegic
MET	mesenchymal-epithelial transition
EMT	epithelial-mesenchymal transition
NC	Negative Control

## References

[1] Siegel R.L., Miller K.D., Jemal A. (2016). Cancer statistics, 2016. CA Cancer J. Clin..

[2] Litwin M.S., Tan H. (2017). The diagnosis and treatment of prostate cancer: A review. JAMA.

[3] Saad F., Fizazi K. (2015). Androgen Deprivation Therapy and Secondary Hormone Therapy in the Management of Hormone-sensitive and Castration-resistant Prostate Cancer. Urology.

[4] Barthel S.R., Hays D.L., Yazawa E.M., Opperman M., Walley K.C., Nimrichter L., Burdick M.M., Gillard B.M., Moser M.T., Pantel K. (2013). Definition of Molecular Determinants of Prostate Cancer Cell Bone Extravasation. Cancer Res..

[5] Julius S., Allegrucci C., Boorjian S.A., Mongan N.P., Persson J.L. (2012). Overcoming Drug Resistance and Treating Advanced Prostate Cancer. Curr. Drug Targets.

[6] Lang L., Shay C., Zhao X., Teng Y. (2017). Combined targeting of Arf1 and Ras potentiates anticancer activity for prostate cancer therapeutics. J. Exp. Clin. Cancer Res..

[7] Chen W.Y., Liu S.Y., Chang Y.S., Yin J.J., Yeh H.L., Mouhieddine T.H., Hadadeh O., Abou-Kheir W., Liu Y.N. (2015). MicroRNA-34a regulates WNT/TCF7 signaling and inhibits bone metastasis in Ras-activated prostate cancer. Oncotarget.

[8] Jiang H., Grenley M.O., Bravo M.J., Blumhagen R.Z., Edgar B.A. (2011). EGFR/Ras/MAPK signaling mediates adult midgut epithelial homeostasis and regeneration in Drosophila. Cell Stem Cell.

[9] Roberts P.J., Der C.J. (2007). Targeting the Raf-MEK-ERK mitogen-activated protein kinase cascade for the treatment of cancer. Oncogene.

[10] Herbst A., Kolligs F.T., Sioud M. (2007). Wnt Signaling as a Therapeutic Target for Cancer. Target Discovery and Validation Reviews and Protocols: Volume 2: Emerging Molecular Targets and Treatment Options.

[11] Lai E.C., Tomancak P., Williams R.W., Rubin G.M. (2003). Computational identification of Drosophila microRNA genes. Genome Biol..

[12] Bartel D.P. (2017). MicroRNAs. Cell.

[13] Miranda K.C., Huynh T., Tay Y., Ang Y.S., Tam W.L., Thomson A.M., Lim B., Rigoutsos I. (2006). A Pattern-Based Method for the Identification of MicroRNA Binding Sites and Their Corresponding Heteroduplexes. Cell.

[14] Lewis B.P., Burge C.B., Bartel D.P. (2005). Conserved Seed Pairing, often Flanked by Adenosines, Indicates that Thousands of Human Genes are MicroRNA Targets. Cell.

[15] Lu J., Getz G., Miska E.A., Alvarez-Saavedra E., Lamb J., Peck D., Sweet-Cordero A., Ebert B.L., Mak R.H., Ferrando A.A. (2005). MicroRNA expression profiles classify human cancers. Nature.

[16] Liu C., Kelnar K., Liu B., Chen X., Calhoun-Davis T., Li H., Patrawala L., Yan H., Jeter C., Honorio S. (2011). The microRNA miR-34a inhibits prostate cancer stem cells and metastasis by directly repressing CD44. Nat. Med..

[17] Gandellini P., Folini M., Longoni N., Pennati M., Binda M., Colecchia M., Salvioni R., Supino R., Moretti R., Limonta P. (2009). miR-205 Exerts Tumor-Suppressive Functions in Human Prostate through Down-regulation of Protein Kinase Cε. Cancer Res..

[18] Takeshita F., Patrawala L., Osaki M., Takahashi R., Yamamoto Y., Kosaka N., Kawamata M., Kelnar K., Bader A.G., Brown D. (2010). Systemic Delivery of Synthetic MicroRNA-16 Inhibits the Growth of Metastatic Prostate Tumors via Downregulation of Multiple Cell-cycle Genes. Mol. Ther..

[19] He L., He X., Lim L.P., de Stanchina E., Xuan Z., Liang Y., Xue W., Zender L., Magnus J., Ridzon D. (2007). A microRNA component of the p53 tumour suppressor network. Nature.

[20] Ellsworth R.E., Vertrees A., Love B., Hooke J.A., Ellsworth D.L., Shriver C.D. (2008). Chromosomal Alterations Associated with the Transition from In Situ to Invasive Breast Cancer. Ann. Surg. Oncol..

[21] Rasio D., Negrini M., Manenti G., Dragani T.A., Croce C.M. (1995). Loss of Heterozygosity at Chromosome 11q in Lung Adenocarcinoma: Identification of Three Independent Regions. Cancer Res..

[22] Dahiya R., McCarville J., Lee C., Hu W., Kaur G., Carroll P., Deng G. (1997). Deletion of chromosome 11p15, p12, q22, q23-24 loci in human prostate cancer. Int. J. Cancer.

[23] Navarro F., Lieberman J. (2015). miR-34 and p53: New Insights into a Complex Functional Relationship. PLoS ONE.

[24] Ebner O.A., Selbach M. (2014). Quantitative Proteomic Analysis of Gene Regulation by miR-34a and miR-34c. PLoS ONE.

[25] Prokopi M., Kousparou C.A., Epenetos A.A. (2015). The Secret Role of microRNAs in Cancer Stem Cell Development and Potential Therapy: A Notch-Pathway Approach. Front. Oncol..

[26] Hermeking H. (2012). MicroRNAs in the p53 network: Micromanagement of tumour suppression. Nat. Rev. Cancer.

[27] Tarasov V., Jung P., Verdoodt B., Lodygin D., Epanchintsev A., Menssen A., Meister G., Hermeking H. (2007). Differential Regulation of microRNAs by p53 Revealed by Massively Parallel Sequencing: miR-34a is a p53 Target That Induces Apoptosis and G1-arrest. Cell Cycle.

[28] Rokavec M., Li H., Jiang L., Hermeking H. (2014). The p53/miR-34 axis in development and disease. J. Mol. Cell Biol..

[29] Hagman Z., Haflidadottir B.S., Ansari M., Persson M., Bjartell A., Edsjö A., Ceder Y. (2013). The tumour suppressor miR-34c targets MET in prostate cancer cells. Br. J. Cancer.

[30] Aalinkeel R., Nair M.P., Sufrin G., Mahajan S.D., Chadha K.C., Chawda R.P., Schwartz S.A. (2004). Gene Expression of Angiogenic Factors Correlates with Metastatic Potential of Prostate Cancer Cells. Cancer Res..

[31] Majid S., Dar A.A., Saini S., Shahryari V., Arora S., Zaman M.S., Chang I., Yamamura S., Tanaka Y., Chiyomaru T. (2012). miRNA-34b Inhibits Prostate Cancer through Demethylation, Active Chromatin Modifications, and AKT Pathways. Clin. Cancer Res..

[32] Hagman Z., Larne O., Edsjö A., Bjartell A., Ehrnström R.A., Ulmert D., Lilja H., Ceder Y. (2010). miR-34c is downregulated in prostate cancer and exerts tumor suppressive functions. Int. J. Cancer.

[33] Chaffer C.L., Weinberg R.A. (2011). A Perspective on Cancer Cell Metastasis. Science.

[34] Bellomo C., Caja L., Moustakas A. (2016). Transforming growth factor β as regulator of cancer stemness and metastasis. Br. J. Cancer.

[35] Folkman J., Klagsbrun M. (1987). Angiogenic factors. Science.

[36] Miguchi M., Hinoi T., Shimomura M., Adachi T., Saito Y., Niitsu H., Kochi M., Sada H., Sotomaru Y., Ikenoue T. (2016). Gasdermin C Is Upregulated by Inactivation of Transforming Growth Factor β Receptor Type II in the Presence of Mutated Apc, Promoting Colorectal Cancer Proliferation. PLoS ONE.

[37] Tian A., Ma H., Zhang R., Tan W., Wang X., Wu B., Wang J., Wan C. (2015). Interleukin17A Promotes Postoperative Cognitive Dysfunction by Triggering β-Amyloid Accumulation via the Transforming Growth Factor-β (TGFβ)/Smad Signaling Pathway. PLoS ONE.

[38] Choi Y.J., Lin C.-P., Ho J.J., He X., Okada N., Bu P., Zhong Y., Kim S.Y., Bennett M.J., Chen C. (2011). miR-34 miRNAs provide a barrier for somatic cell reprogramming. Nat. Cell Biol..

[39] Forno I., Ferrero S., Russo M.V., Gazzano G., Giangiobbe S., Montanari E., Del Nero A., Rocco B., Albo G., Languino L.R. (2015). Deregulation of MiR-34b/Sox2 Predicts Prostate Cancer Progression. PLoS ONE.

[40] Lan H.Y. (2011). Diverse Roles of TGF-β/Smads in Renal Fibrosis and Inflammation. Int. J. Biol. Sci..

[41] Yang H., Fang F., Chang R., Yang L. (2013). MicroRNA-140-5p suppresses tumor growth and metastasis by targeting transforming growth factor β receptor 1 and fibroblast growth factor 9 in hepatocellular carcinoma. Hepatology.

[42] Wu K., Zhao Z., Ma J., Chen J., Peng J., Yang S., He Y. (2017). Deregulation of miR-193b affects the growth of colon cancer cells via transforming growth factor-β and regulation of the SMAD3 pathway. Oncol. Lett..

